# Does carbon monoxide treatment alter cytokine levels after endotoxin infusion in pigs? A randomized controlled study

**DOI:** 10.1186/1476-9255-5-13

**Published:** 2008-08-07

**Authors:** Anna-Maja Åberg, Pernilla Abrahamsson, Göran Johansson, Michael Haney, Ola Winsö, Jan Erik Larsson

**Affiliations:** 1 Division of Anaesthesiology and Intensive Care Medicine, Department of Surgical and Perioperative Sciences, Umeå University Hospital, Umeå, Sweden

## Abstract

**Background:**

Carbon monoxide (CO) has recently been suggested to have anti-inflammatory properties, but data seem to be contradictory and species-specific. Thus, in studies on macrophages and mice, pretreatment with CO attenuated the inflammatory response after endotoxin exposure. On the other hand, human studies showed no effect of CO on the inflammatory response. Anti-inflammatory efficacy of CO has been shown at concentrations above 10% carboxyhaemoglobin. This study was undertaken to elucidate the possible anti-inflammatory effects of CO at lower CO concentrations.

**Methods:**

Effects of CO administration on cytokine (TNF-alpha, IL-6, IL-1beta and IL-10) release were investigated in a porcine model in which a systemic inflammatory response syndrome was induced by endotoxin infusion. Endotoxin was infused in 20 anaesthetized and normoventilated pigs. Ten animals were targeted with inhaled CO to maintain 5% COHb, and 10 animals were controls.

**Results:**

In the control group, mean pulmonary artery pressure increased from a baseline value of 17 mmHg (mean, n = 10) to 42 mmHg (mean, n = 10) following 1 hour of endotoxin infusion. Similar mean pulmonary artery pressure values were found in animals exposed to carbon monoxide. Plasma levels of all of the measured cytokines increased in response to the endotoxin infusion. The largest increase was observed in TNF-alpha, which peaked after 1.5 hours at 9398 pg/ml in the control group and at 13395 pg/ml in the carbon monoxide-exposed group. A similar peak was found for IL-10 while the IL-6 concentration was maximal after 2.5 hours. IL-1beta concentrations increased continuously during the experiment. There were no significant differences between carbon monoxide-exposed animals and controls in any of the measured cytokines.

**Conclusion:**

Our conclusion is that 5% COHb does not modify the cytokine response following endotoxin infusion in pigs.

## Background

Carbon monoxide (CO) is recognized as a toxic gas in humans, originating from tobacco smoke, car exhaust and fire. CO bound to haemoglobin (Hb) can lead to injury related to impaired oxygen delivery, since the affinity of Hb for CO is much greater than for oxygen. CO also interferes with cellular respiration through the electron transport chain by inhibition of cytochrome c oxidase. However, some studies suggest that CO also has positive biological effects such as a vasodilative action [1,2]. Many in vitro studies, as well as studies in rodents postulate anti-inflammatory effects of CO [3-7]. A conflicting lack of effect of CO was found in humans after endotoxin exposure, where no protective or anti-inflammatory effects were demonstrated [8].

Our hypothesis was that a low dose of CO has protective anti-inflammatory effects during sepsis. We aimed to test this using a model of endotoxin-induced systemic inflammation in pigs. Further, we aimed to test this at CO levels below concentrations that may be toxic.

## Methods

The study was approved by the Animal Experimental Ethics Committee and performed in accordance with the NIH Institutional animal care and use committee guidebook. A total of 20 female pigs weighing 23–40 kg were used. They were delivered from the breeder to the University stable and kept overnight.

### Anaesthesia

For premedicination, a mixture of ketamine 10 mg/kg (Ketalar^®^, Pfizer, Morris Plains, New Jersey, USA), azaperone 4 mg/kg (Stresnil^®^, Janssen-Cilag, Neuss, Germany) and atropine sulphate 0.05 mg/kg (Atropin, NM Pharma, Stockholm, Sweden) was given intramuscularly. Anaesthesia was induced by an intravenous bolus dose of 10 mg/kg sodium pentobarbital (Pentobarbitalnatrium, Apoteksbolaget, Stockholm, Sweden). Infusion of fentanyl (Fentanyl, Braun, Melsungen, Germany) 20 μg/kg/h, midazolam (Dormicum, Roche, Basel, Switzerland) 0.3 mg/kg/h and sodium pentobarbital 5 mg/kg/h was used for maintenance of anaesthesia. The animals were tracheotomized (7.0 OP endotracheal tube, Rusch, Kernen, Germany) and mechanically ventilated with air containing 30% oxygen (Evita 4, Dräger, Germany). The ventilator was set to give a positive end-expiratory pressure of 3 cm H_2_O. Ventilation was adjusted to obtain normoventilation, as determined by the goal of P_a_CO_2 _levels between 4.5 and 5.5 kPa, as measured with intermittent arterial blood gas analyses (ABL5 autoanalyzer, Radiometer, Copenhagen, Denmark). During the protocol, the fraction of inspired oxygen (FiO_2_) was adjusted to avoid hypoxia (FiO_2 _varied between 30–100%), as measured by the arterial oxygen saturation (S_a_O_2_) of haemoglobin and the Hb concentration (OSM3 hemoximeter, Radiometer, Denmark). A S_a_O_2 _of more than 90% and a Hb concentration of more than 90 g/l were considered sufficient for this purpose. One litre of Ringer's acetate was given to the animals during the first hour of the preparation and stabilisation period, and was followed by an infusion that started at 15 ml/kg/h and was increased during the day to maintain normovolemia, as determined by the goal to achieve a CVP between 5 and 10 mmHg.

### Instrumentation

All vascular catheterisations were conducted by vessel cutdowns in the neck. An arterial catheter was placed in a small neck artery. A central venous catheter was inserted in the external jugular vein. A 7F, 4-lumen, balloon-tipped pulmonary artery catheter (Optimetrix, Abbot Inc. Illinois, USA) was placed to an occlusion position in the pulmonary vascular tree, where the balloon was deflated and the catheter secured. Measurements included heart rate (HR), mean arterial pressure (MAP), central venous pressure (CVP) and mean pulmonary arterial pressure (MPAP). Cardiac output was measured by thermodilution with 5 ml iced saline as indicator (WTI, Wetenskappwlijk, Technische Instituut, Rotterdam, The Netherlands). All pressures were measured using fluid filled catheters and pressure transducers (Ohmeda Inc., USA) at the mid-axillary level. HR and all pressure measurements were continuously recorded using a computer based multi-channel signal acquisition and analysis system (Acqknowledge, Biopac systems Inc., CA, USA).

### Experimental Protocol

The animals were randomized following pre-medication to receive CO or not until equal numbers of CO-infused and control pigs were obtained. The treatment was open to all personnel performing the experiment. One hour after the preparation, CO (5% in nitrogen) was administrated to the low-pressure circuit of the ventilator. First, a bolus of CO was given with the goal to obtain 5% COHb in the blood, as determined by hemoxiometry (OSM3 hemoximeter, Radiometer, Denmark). This was followed by delivery of CO at a flow rate of 4–50 ml/min throughout the protocol to match a predicted clearance of 25 ml/min [9] and to maintain a stable CO level, as measured by COHb concentrations. Ten animals were used as controls and were not given CO. Two hours after the preparation, endotoxin (lipopolysaccharides from Escherichia coli, 0111:B4, Sigma, USA) was infused intravenously, beginning at 0.05 μg/kg/h and reaching 0.25 μg/kg/h after 30 minutes, which was maintained during the remaining protocol. This infusion rate aimed at a total dose of 1.175 μg/kg to each animal. The endotoxin dose was not adjusted when the animals demonstrated respiratory or circulatory dysfunction. Blood samples were taken every 30 minutes. The total protocol time was 6 hours, including 5 hours of endotoxin infusion.

### Analysis

A total of 13 blood samples were collected from each animal. All arterial and mixed venous blood samples were analysed immediately for P_a_O_2_, P_a_CO_2 _(ABL5 auto analyzer, Radiometer, Denmark), Hb and Hb-saturation (OSM3). Double samples of all 13 arterial blood samples were collected in gas tight tubes and kept at 4°C until they were analysed for CO. CO analysis was performed using gas chromatography (GC) with a nickel catalyst and flame ionization detection (HP 5790A, Agilent Technologies Sweden AB, Stockholm, Sweden), as described elsewhere [10]. The concentration from the gas chromatograph was also calculated to COHb fraction using the transformation [9]:

$$\text{COHb}=\frac{\text{C}•64400}{4•\text{Hb}}$$

Where C is the CO concentration expressed in M, COHb is the carboxyhaemoglobin fraction, Hb is the haemoglobin concentration (g/l), 64400 is the molecular mass of haemoglobin in mammals and the constant 4 represents the four binding sites of haemoglobin to carbon monoxide.

Ten of the arterial blood samples were collected in EDTA tubes (BD Vacutainer^®^, NJ, USA) and centrifuged at 4°C, 3000 G, for 20 minutes. The plasma was collected and stored at -80°C. These plasma samples were analysed for cytokines (TNF-a, IL-6, IL-10 and IL-1beta) using ELISA with porcine antibody kits (R&D Systems Inc., USA) in accordance with the instructions delivered by the manufacturer. The absorbance was read on a spectrophotometer (Labsystems Multiskan MS, Triad Scientific Inc., USA).

### Statistical analysis

A two-sample power analysis was performed using data from an in vivo study in mice where the difference in TNF-alpha concentration between CO exposed animals and controls was 30% in the group exposed to 10 ppm CO [5]. The standard deviation was calculated using SEM values presented in the article and n = 7. Based on these results, an experimental design with 10 animals in each group would give a power of 99%, with an alfa p-level of 0.05 and a beta p-level of 0.007. For each measurement point in each group, the one-sample Kolmogorov-Smirnov test for normality was performed (SPSS 12.0, SPSS Inc. Chicago, USA) for the parameters; MPAP, CO concentrations and plasma cytokine concentrations. No significant differences from normality were found at a p-level of 0.05, indicating that these data were normally distributed. The effect of CO on MPAP, plasma cytokine concentrations and CO concentrations were analysed by SPSS 12.0 (SPSS Inc., Chicago, USA) using mixed between-within subjects analysis of variance for repeated measures (ANOVA). A p-value of less than 0.05 was considered to be a statistically significant difference.

## Results

Seventeen of 20 animals completed the endotoxin protocol and all measurement points. One animal in the CO group died during the 4^th ^hour of endotoxin infusion resulting in missing values at 270 and 300 minutes. Two animals from the control group died before the protocol was completed, one after 2 hours of endotoxin infusion and one after 3.5 hours of endotoxin infusion.

### General circulatory and blood gas data

**Figure 1:**
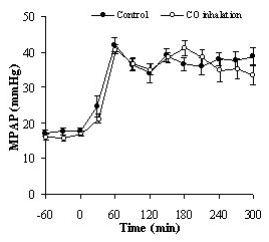
**Mean pulmonary artery pressure in pigs after endotoxin induced systemic inflammation**. Values are represented as means ± SEM for CO treated animals (open circles, n = 10 except at 270 and 300 min where n = 9) and controls (closed circles, n = 10 except at 150, 180 and 210 min where n = 9 and at 240, 270 and 300 min where n = 8). Endotoxin was administered (0.05 μg/kg/h) just after time 0, reaching maximum infusion rate (0.25 μg/kg/h) at 30 min. CO was administrated just after time -60 min. No significant difference between the groups (ANOVA F(1, 9) = 0.158).

General circulatory and blood gas data from selected measurement points are presented in Table 1. MPAP increased to a first peak of almost 50 mmHg after 60 minutes of endotoxin infusion and reached a second peak at approximately 180 minutes indicating a severe systemic inflammatory response. There were no differences in this pattern related to CO (Figure 1). Cardiac output decreased during the protocol (Table 1). Levels of P_a_CO_2 _increased during the experimental procedure, but remained within the normocapnic range.

**Table 1:** Circulatory and respiratory data from pigs during endotoxin infusion.

		**-60 (min)**	**0 (min)**	**60 (min)**	**120 (min)**	**210 (min)**	**300 (min)**
	*group*	*mean ± sem*	*mean ± sem*	*mean ± sem*	*mean ± sem*	*mean ± sem*	*mean ± sem*
**HR**	*Control*	102 ± 7	90 ± 4	93 ± 6	96 ± 8	91 ± 8 a	97 ± 8 b
(bpm)	*CO*	103 ± 4	94 ± 7	88 ± 4	79 ± 4	88 ± 5	92 ± 8 b
**MAP**	*Control*	101 ± 5	95 ± 4	88 ± 5	91 ± 8	93 ± 11 a	95 ± 6 b
(mmHg)	*CO*	100 ± 4	89 ± 3	84 ± 5	94 ± 3	89 ± 7	86 ± 7 a
**CVP**	*Control*	3 ± 0.6	4 ± 0.6	7 ± 0.6	8 ± 0.8	8 ± 0.8 a	6 ± 0.6 b
(mmHg)	*CO*	4 ± 0.7	4 ± 0.8	6 ± 0.8	7 ± 0.7	6 ± 0.7	7 ± 0.6 a
**Cardiac**	*Control*	5.0 ± 0.4 a	4.8 ± 0.4	3.7 ± 0.4	4.1 ± 0.4 a	2.9 ± 0.3 a	3.5 ± 0.5 b
**output **(l/min)	*CO*	5.8 ± 0.3	5.2 ± 0.3	3.8 ± 0.2 a	3.7 ± 0.3	2.9 ± 0.2 a	3.0 ± 0.2 a
**P_a_CO**_2_	*Control*	4.5 ± 0.3 b	5.0 ± 0.2 a	5.3 ± 0.2 a	5.8 ± 0.1 b	5.9 ± 0.3 c	5.9 ± 0.3 b
(kPa)	*CO*	4.5 ± 0.2	4.9 ± 0.1	5.4 ± 0.2	5.7 ± 0.2	6.0 ± 0.3	6.2 ± 0.9 a
**P_a_O**_2_	*Control*	19.3 ± 0.7 b	18.3 ± 0.4 a	29.2 ± 3.0 a	28.3 ± 6.3 b	25.0 ± 5.7 c	19.4 ± 3.6 b
(kPa)	*CO*	20.4 ± 0.5	20.0 ± 0.7	38.2 ± 5.7	38.7 ± 5.8	22.8 ± 5.1	28.5 ± 5.8 a
**Hb**	*Control*	92 ± 1.9 a	89 ± 1.5	95 ± 2.7	100 ± 2.2	108 ± 1.2 a	103 ± 2.2 b
(g/l)	*CO*	93 ± 2.8	88 ± 2.1	91 ± 1.6	101 ± 1.8	108 ± 2.9	105 ± 3.5 a
**FiO**_2_	*Control*	30 ± 0	30 ± 0	55 ± 5.8	62 ± 8.0	68 ± 7.0	70 ± 6.6
(%)	*CO*	30 ± 0	30 ± 0	56 ± 5.9	64 ± 8.4	78 ± 8.3	81 ± 7.5

Administration of CO began 1 hour before the endotoxin infusion was started, whereas control animals received endotoxin infusion but no CO inhalation. Values are presented as means ± SEM, n = 10 in each group (Control and CO), except otherwise stated (a, b, c; n = 9, 8, 7 respectively, as indexed). Endotoxin was administered (0.05 μg/kg/h) just after time 0, reaching maximum infusion rate (0.25 μg/kg/h) at 30 min. CO was administrated just after time -60 min.

### Carbon Monoxide

**Figure 2:**
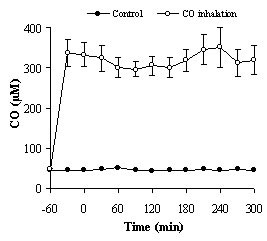
**Carbon monoxide concentrations in the two groups after endotoxin induced systemic inflammation in pigs**. Values are represented as means ± SEM, for CO treated animals (open circles, n = 10 except at 270 and 300 min where n = 9) and controls (closed circles, n = 10 except at 150, 180 and 210 min where n = 9 and at 240, 270 and 300 min where n = 8). Endotoxin was administered (0.05 μg/kg/h) just after time 0, reaching maximum infusion rate (0.25 μg/kg/h) at 30 min. CO was administrated just after time -60 min.

Results from blood analyses of CO concentrations are presented in Figure 2, where 250 μM corresponds to approximately 5% COHb according to the transformation. The control group showed very low CO concentrations (approximately 50 μM) with small inter individual variability. CO administration to 10 animals resulted in steady CO levels throughout the protocol, where 250 μM in blood was the target concentration.

### Cytokines

**Figure 3:**
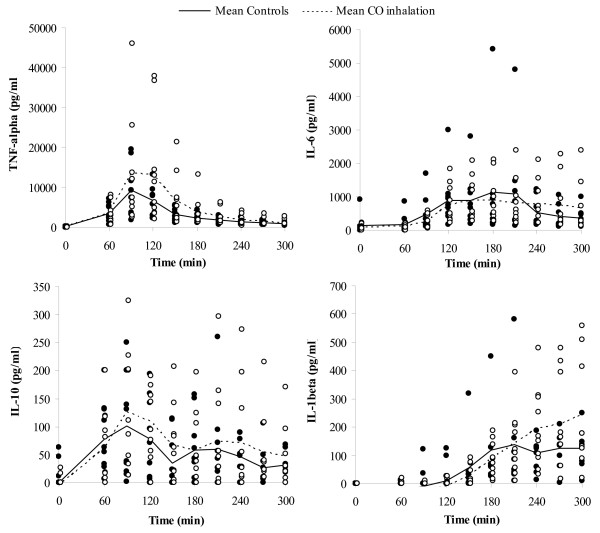
**Plasma cytokine concentrations in pigs after endotoxin-induced systemic inflammation with or without CO treatment**. Values are presented as individual measurements for CO treated animals (open circles) and controls (closed circles). A dotted (CO group) and solid (controls) line represents means for the two groups (n = 10 except for the CO-group at 270 and 300 min where n = 9 and for controls at 150, 180 and 210 min where n = 9 and at 240, 270 and 300 min where n = 8). Endotoxin was administered (0.05 μg/kg/h) just after time 0, reaching maximum infusion rate (0.25 μg/kg/h) at 30 min. No significant differences were detected between the groups for any of the cytokines (TNF: ANOVA *F*(1, 8) = 1.074, IL-6: ANOVA *F*(1, 8) = 0.892, IL-10: ANOVA *F*(1, 8) = 1.347, IL-1beta: ANOVA *F*(1, 8) = 1.716).

Plasma cytokine measurements are shown in Figure 3. TNF-alpha concentrations increased after 60 minutes of endotoxin infusion and decreased after approximately 150 minutes. There was no difference between the groups regarding TNF-alpha concentrations. There was a large variation between individuals, especially at peak levels. Two animals in the CO-treated group had much higher TNF-alpha peak concentrations than the others. Concentrations of IL-6 increased in response to endotoxin infusion, with a peak at 150 minutes followed by a decrease, but not to baseline levels. The two animals with extreme TNF-alpha levels also had relatively high IL-6 concentrations. The individuals with the highest IL-6 concentrations were in the control group and died before the protocol was completed. There was no statistically significant difference in IL-6 concentrations between the groups. The IL-10 concentration peaked at 90 minutes after which it quickly decreased to near baseline levels and no difference was observed between groups. IL-1beta increased continuously during the protocol with the highest levels after 5 hours of endotoxin infusion. One of the animals with the highest IL-6 concentrations also had the highest IL-1beta concentrations. This animal died before the protocol was completed. IL-1beta concentrations were not statistically significant different in CO-treated animals compared with controls.

## Discussion

We were unable to show that administration of CO had any effect on cytokine release during endotoxin-induced inflammatory response. Pro-inflammatory cytokines (TNF-alpha, IL-6 and IL-1beta) were neither attenuated in CO-treated animals, nor did the anti-inflammatory cytokine (IL-10) increase. These results were unexpected and contrasted to findings in an endotoxin mouse model, where lower TNF-alpha and IL-1beta and higher IL-10 levels in CO-treated animals compared with controls were found [5]. In the present study, 3 animals died before completing the whole duration of the protocol, 2 control animals and 1 animal in the CO exposed group. These animals are not included in the statistical calculations due to the limitations of ANOVA, resulting in the fact that the animals that may have had the most powerful inflammatory response may have been excluded from comparison. Analysis of the data shows that the 3 animals that died before completing the protocol did not have the highest TNF-alpha or IL-10 concentrations. However, the highest IL-1beta concentration was found in a control animal that died following 4 hours of endotoxin exposure. The 2 animals from the control group that died had the highest IL-6 concentrations. If these 3 animals would have survived and been included the statistical analysis, this could imply a difference in the interpretation of the IL-6 and IL-1beta concentrations. However, these missing data do not have any effect on the conclusion regarding TNF-alpha and IL-10 response which remains contradictory to the mouse study [5]. Published data on inflammatory effects of CO in pigs is limited to only one other study, where higher levels of TNF-alpha were found in CO-treated animals compared with controls [11]. It was concluded [11] that although the TNF-alpha levels were higher in the CO treated group, CO ameliorated several of the acute pathological changes. They also found a suppression of IL-1beta in the CO-treated group, resulting in a significantly higher level of IL-1beta in the control group. This is in contrast to our findings, which show no differences in IL-1beta concentrations as a result of CO administration. One explanation for this conflicting result could be that the other study [11] only included 4 animals in each group. In a study in man, where CO was administered before a bolus of endotoxin was injected, there were no differences in plasma cytokines (TNF-alpha, IL-6, IL-8, IL-10), cytokine mRNA (IL-1 alpha, IL-1 beta), heart rate, MAP or SpO_2 _when the CO-treated group was compared with controls [8]. These clinical findings also support the interpretation that CO does not help to improve the inflammatory response after endotoxin infusion. Our interpretation of previous studies together with our findings is that CO may have an anti-inflammatory effect in mice but not in humans or pigs.

The cytokine levels following endotoxin infusion in our study were high, and individual TNF-alpha levels were found up to 46000 pg/ml. In comparison, other endotoxin studies in pigs reported maximum levels of TNF-alpha of 3500 pg/ml [11], 4000 pg/ml [12], 9000 pg/ml [13] or 20000 pg/ml [14], respectively. The cytokine response for TNF-alpha, IL-6 and IL-10 following endotoxin infusion shows the same pattern over time in our study as has been observed by others [14], but the IL-1beta response was different. Our findings show an increase in IL-1beta concentration during endotoxin infusion, whereas the other study [14] showed no change in IL-1beta response.

In order to further evaluate possible anti-inflammatory effects of CO, we have used a porcine model of human sepsis. Pig sensitivity to endotoxin and tissue antigenicity has been found to be similar to humans [15]. Furthermore, pigs also have similar cardiac anatomy and physiology as humans [16]. The endotoxin infusion model appeared to provide a highly stable and predictable circulatory and pathophysiological state for our study, as demonstrated by a consistent biphasic MPAP pattern. The endotoxin infusion rate was 0.25 μg/kg/h, corresponding to a total dose of 1.175 μg/kg. The same dose has been used in one other study investigating central haemodynamics [17]. This is a low dose compared with other pig studies [11,13]. Since there are different serotypes of endotoxin, there may be a wide range of potency. Compared with other studies, which have employed the same lipopolysaccharide serotype as in the present study (0111:B4), we still have a low dose of endotoxin. Endotoxin dosing regimens for the same serotype have been the following; a bolus of 100 μg/kg [12], a bolus of 75 μg/kg [18], and an infusion of a total dose of 250 μg/kg [19]. Different batches of endotoxin probably have different potency. Also, different breeds of pigs probably have different sensitivity to endotoxin. The MPAP levels in our study were high in comparison with other authors [11,20] or similar [21]. This acute increase in MPAP associated with endotoxin administration (Figure 1) was close or similar to levels found in cardiovascular decompensation. Given this perspective of wide variation in endotoxin dosing for pig sepsis models, our interpretation is that the low endotoxin dose in our study resulted in large cytokine release as well as high MPAP levels, indicating a massive systemic inflammatory activation.

The administration rate of CO in this study was chosen with the aim to quickly achieve constant blood CO levels and to avoid toxic effects. In contrast to a fixed CO dose, the rate of delivery was modulated in order to maintain relatively constant blood CO concentrations. An increase in the CO administration rate was necessary during the experiment, which we interpret as a result of reduced pulmonary gas exchange due to the severe inflammatory response. Constant CO levels were achieved, which is a strength in this study compared to other studies, in which the CO concentration decreased during the experiment [8,11] or never was measured [5]. The chosen target concentration of CO (5% COHb) in the present study was determined to be a clinically relevant dose, since higher doses may induce toxic symptoms. A CO concentration of 20% in the blood may lead to unconsciousness [22,23]. Negative effects on performance during exercise after carbon monoxide inhalation in healthy men can be seen at CO levels from 4.8% COHb [24]. Studies on patients with angina pectoris show that carbon monoxide at levels from 2.7% to 4.5% COHb shortens the time to pain during exercise and also induces a longer duration of pain [25-27]. Performance during exercise in patients with chronic anaemia is reduced at 2.0% COHb [28]. The relation between CO dose and inflammatory response may be important. Effects in pigs have been described at 10–12% COHb [11], but no effects in humans have been reported at 7% COHb [8]. If the previously suggested anti-inflammatory effect of CO is found at these higher CO concentrations, this may imply that the therapeutic potential of CO is limited due to the risk of toxic side effects.

An important consideration regarding the animal model is that the affinity of Hb for CO is dependent upon the studied animal species. For example, mouse Hb has lower affinity for CO compared with human Hb [8]. Pig Hb has lower affinity for CO than some other mammals, e.g. rat and hamster [29]. A lower affinity of Hb for CO could result in a higher unbound or free fraction of CO, eliciting a greater biological response at similar COHb fractions. Elimination time for CO may also vary in different species, as well as by differences in oxygenation. It has been shown that the affinity of Hb for CO increases at low oxygen tension [30]. All of this has to be considered when evaluating the proper dose of CO. This also points out why it is of great importance to measure CO concentrations in the studied subjects, in contrast to measurements of ambient or inhaled CO levels.

## Conclusion

In summary, no clear effects of CO on the systematic inflammatory process were shown in this study conducted in endotoxin administered pigs, as evaluated by measured concentrations of plasma cytokines (TNF-alpha, IL-6, IL-1beta and IL-10). The model was characterised by massive inflammation and a stable and controlled CO level. We conclude that 5% COHb in the blood does not appear to demonstrate any potential therapeutic effects on the modulation of systemic inflammation in this porcine model.

## Competing interests

The authors declare that they have no competing interests.

## Authors' contributions

AMÅ participated in the design of the study, the practical work, the result discussion the statistical calculations and writing the manuscript. PA participated in the practical work, the result discussion and the revision of the manuscript. GJ participated in the practical work, the statistical calculations, the result discussion and the revision of the manuscript. MH participated in the practical work, the result discussion and helped to draft the manuscript. OW participated in the design of the study, the result discussion, revision of the manuscript and financial support. JEL participated in the design of the study, the practical work, the result discussion, the statistical calculations and in writing the manuscript. All authors (AMÅ, PA, GJ, MH, OW and JEL) have read and approved the final manuscript.
